# Spinal circuits can accommodate interaction torques during multijoint limb movements

**DOI:** 10.3389/fncom.2014.00144

**Published:** 2014-11-11

**Authors:** Thomas Buhrmann, Ezequiel A. Di Paolo

**Affiliations:** ^1^Department of Logic and Philosophy of Science, IAS-Research Centre for Life, Mind and Society, UPV/EHU, University of the Basque CountrySan Sebastian, Spain; ^2^Ikerbasque, Basque Foundation for ScienceBilbao, Spain; ^3^Centre for Computational Neuroscience and Robotics, University of SussexBrighton, UK

**Keywords:** motor control, interaction torques, intersegmental dynamics, spinal circuits, internal model, intralimb coordination, equilibrium-point hypothesis

## Abstract

The dynamic interaction of limb segments during movements that involve multiple joints creates torques in one joint due to motion about another. Evidence shows that such interaction torques are taken into account during the planning or control of movement in humans. Two alternative hypotheses could explain the compensation of these dynamic torques. One involves the use of internal models to centrally compute predicted interaction torques and their explicit compensation through anticipatory adjustment of descending motor commands. The alternative, based on the equilibrium-point hypothesis, claims that descending signals can be simple and related to the desired movement kinematics only, while spinal feedback mechanisms are responsible for the appropriate creation and coordination of dynamic muscle forces. Partial supporting evidence exists in each case. However, until now no model has explicitly shown, in the case of the second hypothesis, whether peripheral feedback is really sufficient on its own for coordinating the motion of several joints while at the same time accommodating intersegmental interaction torques. Here we propose a minimal computational model to examine this question. Using a biomechanics simulation of a two-joint arm controlled by spinal neural circuitry, we show for the first time that it is indeed possible for the neuromusculoskeletal system to transform simple descending control signals into muscle activation patterns that accommodate interaction forces depending on their direction and magnitude. This is achieved without the aid of any central predictive signal. Even though the model makes various simplifications and abstractions compared to the complexities involved in the control of human arm movements, the finding lends plausibility to the hypothesis that some multijoint movements can in principle be controlled even in the absence of internal models of intersegmental dynamics or learned compensatory motor signals.

## 1. Introduction

Human multijoint reaching movements are characterized by invariants such as straight hand paths and bell-shaped velocity profiles (Morasso, 1981; Soechting and Lacquaniti, 1981; Atkeson and Hollerbach, 1985). These invariants hold independently of the amplitude, speed and direction of movement, and therefore independently also of the resulting variation in interaction torques that arise in one joint due to motion about another. The absence of a signature of these time-varying torques in observed kinematics indicates that intersegmental dynamics are compensated for in the planning or execution of arm movements (Hollerbach and Flash, 1982).

Evidence suggests that this compensation is not achieved by executing movements with high stiffness (Gomi and Kawato, 1996; Gribble et al., 1998), which would allow muscle forces to dominate over the passively emerging loads. Rather, muscle activity varies with the direction of interaction torques, such that muscle forces acting at one joint (e.g., the shoulder) are dependent on the direction of motion about another joint (elbow), even when the former joint performs the same motion or remains stationary (Cooke and Virji-Babul, 1995; Latash et al., 1995; Gribble and Ostry, 1999; Galloway and Koshland, 2002; Debicki and Gribble, 2005).

Two possibilities could explain the origin of this intralimb coordination strategy. In computational approaches to motor control the brain is assumed to calculate the time-course of forces necessary to perform desired movements using internal models of the body (Kawato, 1999). This implies the prediction of interaction torques and their explicit compensation through anticipatory adjustment of descending motor commands. The speed and accuracy of skilled ballistic movements (such as throwing), during which feedback may be too slow to mediate compensatory signals, suggests the need for such a predictive strategy. Empirical evidence in its support is provided, for example, by experiments showing a correlation between corticospinal excitability and upcoming interaction torques (Gritsenko et al., 2011), or by patients with hemiparesis whose deficits in reaching movements are consistent with a failure to account for intersegmental dynamics (Beer et al., 2000).

An alternative strategy, based on peripheral feedback, is offered by proponents of the equilibrium-point (EP) hypothesis (Feldman, 1966; Feldman and Levin, 1995; Gribble et al., 1998), which suggests that movements are controlled by simple kinematic shifts in the equilibrium position of the limb, while the required forces result from muscle dynamics and spinal circuitry. The hypothesis predicts that descending motor commands need not take into account upcoming interaction torques during multijoint movements. This would imply that intersegmental dynamics need to be accommodated implicitly through either the neural coupling of muscles acting on adjacent joints (via intersegmental spinal circuitry), or through the mechanical properties of the musculoskeletal system itself. Viscoelastic properties of muscles have been shown to counteract interaction forces in some cases (Hirashima et al., 2003). However, since both viscoelastic forces as well as active muscle forces depend on the level of muscle activation, and can therefore not be controlled independently, they are a poor choice for the precise counteraction of intersegmental loads. Indeed, subjects perform skilled movements despite these viscoelastic properties, rather than because of them (Hirashima et al., 2007; Debicki et al., 2011). If the EP hypothesis is to maintain the idea of simple descending motor commands that are “ignorant” of intersegmental dynamics, then motion about different joints needs to be coordinated appropriately through intersegmental neural coupling of spinal circuits. The question we investigate here is whether such a peripheral coordination strategy is possible.

Several observations indicate that feedback compensation of limb dynamics is plausible at least in principle. Firstly, interaction forces arising at one joint are strongly related to the muscle forces applied to another (Gribble and Ostry, 1999; Galloway and Koshland, 2002), and such muscle forces are encoded reliably in the population response of Golgi tendon organs (Mileusnic and Loeb, 2009). Secondly, Ib afferent activity carrying these force-related proprioceptive signals results in widespread modulation of motoneurons innervating muscles acting at adjacent joints (Jankowska et al., 1981). The sensitivity of Ib inhibitory interneurons can be adjusted through input from Ia afferents that carry muscle length and velocity feedback, which allows for precise force regulation throughout a wide range of movements (McCrea, 1992). Though McCrea points out that a hypothesis has yet to emerge that explains this widespread distribution of Ib modulation throughout the limb, it is clear that it would be well suited to play a role in coordinating the simultaneous motion of several joints. Thirdly, functionally deafferented patients have been shown to make systematic movement errors indicative of a failure to counteract interaction forces, demonstrating a functional role for proprioception in the compensation of internal loads (Ghez and Sainburg, 1995; Sainburg et al., 1995). In this experiment the question remains of whether proprioceptive feedback acts in long loops through the CNS, or locally through spinal reflexes. But motion-dependent feedback across spinal segments has also been shown to modulate ongoing limb dynamics in the cat (Smith and Zernicke, 1987; Koshland and Smith, 1989).

Since evidence exists for both predictive and feedback compensation of interaction torques, several authors have suggested that both mechanisms could contribute to the compensation either simultaneously or at different times throughout a movement (Sainburg et al., 1999; Gritsenko et al., 2011). One can also hypothesize that the relative contribution of centrally planned compensation is greater in fast and highly skilled movements, while spinal compensation might be significant in everyday movements such as reaching; or that the spinal contribution is greater early on in development, while being gradually replaced with more precise central corrections acquired by adaptive processes in the CNS. But regardless of the question of when or to what extent it may contribute to a class of movements, the ability and effectiveness of peripheral feedback compensation of interaction torques has yet to be demonstrated. There are, for example, no convincing models of how an EP-approach would work for the peripheral accommodation of interaction torques without recourse to centrally planned compensation.

Our objective in this paper is to fill in a gap in the modeling literature and demonstrate that an EP-based approach can indeed show accommodation of interaction torques. We introduce a model of planar arm movements based on the EP hypothesis, but extended to include spinal reflex dynamics. We show that it is possible to reproduce empirically observed kinematic invariants across a range of directions and magnitudes of interaction torques. Moreover, the model achieves this by transforming simple descending motor commands, derived only from desired movement kinematics, into muscle activation patterns that vary appropriately with upcoming internal loads, independently of their magnitude or other specifics (direction, speed, amplitude) of the movement. Analysis of the model suggests that for some classes of movements the brain may control the motion of limbs as if intersegmental dynamics were absent, while lower level dynamics achieve the necessary coordination locally. For such movements, the brain would not need to rely on internal models of intersegmental dynamics, nor would need to learn a different set of compensatory motor signals for each possible movement.

The biomechanical model employed in this simulation study is deliberately simple. This is because we do not aim primarily at elucidating how exactly, i.e., in quantitative detail, humans compensate for interaction torques. Rather, the aim is to show for the first time that in principle such compensation can be implemented solely on the level of body dynamics and peripheral feedback. We note that intersegmental dynamics, and the problem of accounting for it, not only occurs in human arm movements. It has to be dealt with in movements performed by any (natural or artificial) multijoint system, independent of how it is actuated (whether, for examples, by muscles or motors), as long as movements are executed in a compliant manner. We therefore model a system that structurally is similar enough to certain human arm movements to allow for qualitative comparisons, while abstracting away features that are of little relevance for the question of whether feedback through local spinal circuits is sufficient on its own for the compensation of interaction torques. Specifically, we omit from the model the tendons that connect muscles to the skeleton (which in the case of the upper arm are sufficiently short and inelastic to justify this simplification, see Section 2.2 for further details); and chose to also omit biarticular muscles. The latter choice implies that the results obtained from the model, such as certain muscle activation patterns, cannot necessarily be compared directly with those observed in humans (although we will in fact identify certain similarities). It is certainly the case that biarticular muscles, exactly because they span neighboring joints, may have a special role to play in intersegmental dynamics. However, their omission here allows us to disentangle their potential contribution to interaction torque compensation from contributions due to peripheral feedback mechanisms. In fact, we show here that even without such muscles, feedback between local spinal circuits alone is sufficient for such compensation. In this sense, the level of abstraction chosen in our simulations is analogous to other models that are concerned more generally with (1) the problem of interaction torques, such as Hollerbach and Flash (1982), in which the problem is investigated purely on the level of joint actuation, without any reference to the role of muscles; (2) the function of spinal circuitry in movement control, for example Raphael et al. (2010), who investigate a spinal model of comparable complexity for the control of a single wrist joint actuated by four symmetric muscles; or (3) the inference of unknown neural mechanisms or physiological properties underlying movement control, as for example Izquierdo and Beer (2013), in which properties of the neural circuit underlying *C. elegans* klinotaxis are investigated using a minimal neuroanatomical model and a methodology similar to the one employed in the experiments reported here.

While demonstrating the feasibility of spinal compensation of interaction torques in an EP-control framework for one class of movements, we do not claim that this is the mode employed for all types of movements, nor that it is the main role of spinal circuitry. If, when, or to what extent spinal dynamics contribute to the compensation of intersegmental loads is an empirical question that we do not address in the work presented here. The result should also not be counted as an argument against internal models, but rather in favor of the complex and often unintuitive control that can be achieved from the bottom-up.

## 2. Materials and methods

In the following sections we describe in detail the biomechanical model of planar arm movements employed in this study; its control via spinal reflex-like neural networks based on known physiology; the integration of spinal dynamics, proprioceptive feedback, and descending commands at α-MNs according to the equilibrium-point hypothesis; and the optimization procedure used to identify model parameters that enable kinematically realistic multijoint movements subject to varying patterns of interaction torques.

### 2.1. Arm model

**Figure 1:**
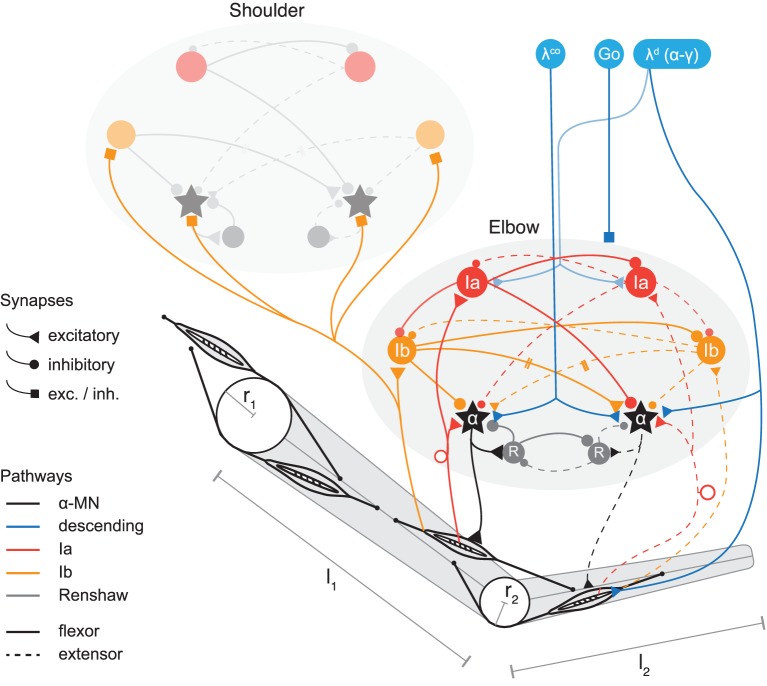
**Model of two-joint planar arm actuated by antagonistic muscles under control of spinal interneurons**. Shown are two spinal circuits, one for each pair of antagonistic muscles. Connections are drawn between interneurons regulating muscles acting on the same joint, as well as those coupling adjacent joints (only one direction is shown for simplicity; the structure of Ib connections between segments is symmetric in the model). Ia pathways are shown in red, Ib pathways in orange, and Renshaw cells in gray. Flexor related circuitry is drawn as solid and extensor as dashed lines. Excitatory synapses are displayed as triangles and inhibitory synapses as disks. Those that during optimization can be of either type are drawn as squares. Three types of signals descend from higher centers (blue). These are: the stretch reflex threshold λ^*d*^ (implying appropriate coordination of α and γ fusimotor drives, see section on threshold control); a coactivation signal λ^*co*^ to the α-MNs, and a GO-signal distributed to all spinal neurons (each receiving this signal via its own weighted connection, not shown here). The topology of the circuits is symmetric, but synaptic strengths can be assigned asymmetrically. The topology is also identical for the two joints, though for clarity some connections of the shoulder joint are omitted in the figure. Muscles wrap around joint capsules of radius *r*_1,2_ and insert into arm segments of lengths *l*_1,2_.

The biomechanical simulation implements the simplest model that allows for the investigation of interaction torques and their compensation, namely a planar arm consisting of two rigid segments connected by hinge joints (see Figures 1, **3**). We will use labels such as “shoulder” and “elbow” for the joints (as well as “flexor” and “extensor” for muscles) in analogy to human physiology, even though aspects of the simplified model may vary in details from their human counterparts. To model the dynamics of the planar arm we use here the formulation by Hollerbach and Flash (1982), which derives joint torques from the arm's kinematics, Newton-Euler equations and d'Alembert's principle. The resulting equations of motion explicitly factor in the contribution of external, inertial, coriolis and centripetal forces:

(1) $$\begin{array}{l} \eta_{2}={\ddot{\theta }}_{1}\left(I_{2}+\frac{m_{2}l_{2}l_{1}}{2}\cos\theta_{2}+\frac{m_{2}l_{2}^{2}}{4}\right)+{\ddot{\theta }}_{2}\left(I_{2}+\frac{m_{2}l_{2}^{2}}{4}\right)\\ \;+\;\frac{m_{2}l_{2}l_{1}}{2}{\dot{\theta }}_{1}^{2}\sin\theta_{2}\\ \eta_{1}={\ddot{\theta }}_{1}\left(I_{1}+I_{2}+m_{2}l_{2}l_{1}\cos\theta_{2}+\frac{m_{1}l_{1}^{2}+m_{2}l_{2}^{2}}{4}+m_{2}l_{1}^{2}\right)\\ \;+\;{\ddot{\theta }}_{2}\left(I_{2}+\frac{m_{2}l_{2}^{2}}{4}+\frac{m_{2}l_{2}l_{1}}{2}\cos\theta_{2}\right)\\ \;-\;\frac{m_{2}l_{2}l_{1}}{2}{\dot{\theta }}_{2}^{2}\sin\theta_{2}-m_{2}l_{2}l_{1}{\dot{\theta }}_{2}{\dot{\theta }}_{1}\sin\theta_{2} \end{array}$$

Here η are torques applied to the joints externally (e.g., by muscles), θ the joint angles, *I* the rigid body inertias, *m* their masses, and *l* their lengths. Subscripts indicate the segment for parameters describing properties of the rigid bodies (1 = upper arm, 2 = lower arm). In the case of torques, angles and their derivatives, subscripts indicate the joint (1 = shoulder, 2 = elbow). We let *m*_1_ = 2.25 kg, *m*_2_ = 1.3 kg, *l*_1_ = 0.33 m and *l*_2_ = 0.32 m, and inertias *I*_*i*_ = (*m*_*i*_ *l*^2^_*i*_)/12 (Karniel and Inbar, 1997). For our analysis of the relative contribution of muscle and interaction torques to the net torques observed in a particular movement, we consider interaction torques to consist of the sum of those terms in the above equations that depend on movement in another joint; e.g., terms depending on $\dot{\theta }$_1_ or $\ddot{\theta }$_1_ in the equation for η_2_.

### 2.2. Muscle model

**Figure 2:**
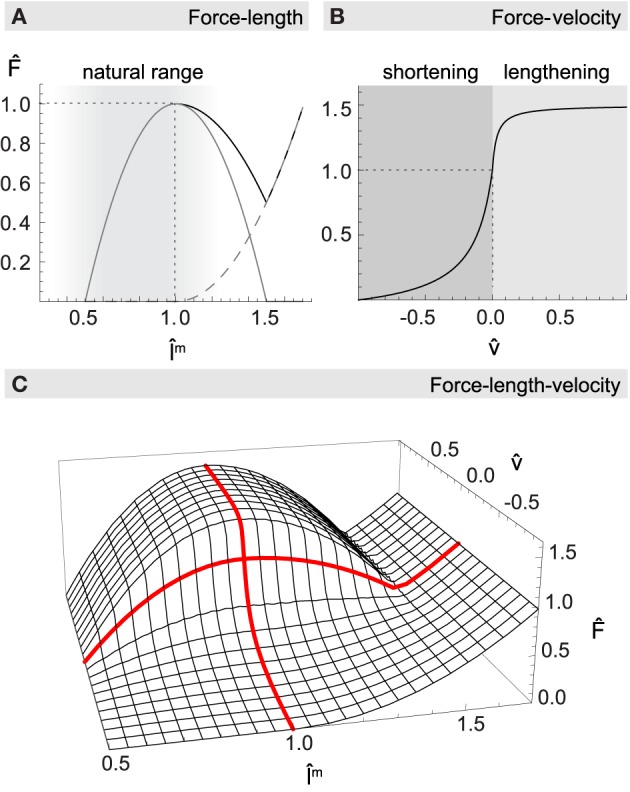
**Normalized muscle force $\hat{F}$ as a function of length $\hat{l}$^*m*^ and velocity $\hat{v}$**. **(A)** The net force-length relationship (black solid line) is formed additively by a passive elasticity resisting lengthening of the muscle (dashed gray line), and a hyperbolic function with maximum at resting length describing the active generation of force (gray solid line). Most muscles of the human upper arm are constrained to the ascending leg of the curve, as indicated by the shaded region. **(B)** The force-velocity relationship describes how force production drops with increasing shortening velocity and increases when actively lengthening. **(C)** Multiplicative combination of muscle length and velocity relationships for maximum activation level *a* = 1. Thick red lines highlight the force-length curve at rest ($\hat{v}$ = 0), and the force-velocity curve when the muscle is at its optimal length ($\hat{l}$^*m*^ = 1).

Each of the two joints is actuated by an antagonistic muscle pair (Figure 1). The lumped muscles are described by a Hill-type model that captures the essential non-linear relationships between muscle length, contraction velocity, and force generation (Zajac, 1989). It consists of three components: an active contractile element in parallel with both a passive elastic spring and a viscous damper. The first component describes a muscle's isometric force generating capability $\hat{F}$_*a*_ as a function of its length and is modeled using the quadratic function

(2) $${\hat{F}}_{a}({\hat{l}}^{m})=1-{\left(\frac{{\hat{l}}^{m}-1}{0.5}\right)}^{2}$$

where *l*^*m*^ is muscle length and variables decorated with the “hat” symbol ( ˆ ) are normalized: $\hat{l}$^*m*^ = *l*^*m*^/*l*^*m*^_0_, $\hat{F}$ = *F*/*F*_*max*_ and *l*^*m*^_0_ the length at which active muscle force reaches its isometric maximum *F*_*max*_. The passive elastic element is described by a quadratic dependence of force $\hat{F}$_*p*_ on muscle extension beyond a given threshold $\hat{l}$^*m*^_*p*_ (Kistemaker et al., 2007):

(3) $${\hat{F}}_{p}({\hat{l}}^{m})=\{\begin{array}{ll} k_{p}{\left({\hat{l}}^{m}-{\hat{l}}_{p}^{m}\right)}^{2} & \text{if }{\hat{l}}^{m}>{\hat{l}}_{p}^{m},\\ 0 & \text{if }{\hat{l}}^{m}\leq {\hat{l}}_{p}^{m} \end{array}$$

where *k*_*p*_ is scaled such that $\hat{F}$_*p*_ = 0.5*F*_*max*_ at the muscle length where $\hat{F}$_*a*_ drops to 0 (Kistemaker et al., 2007) and $\hat{l}$^*m*^_*p*_ = 1 (Zajac, 1989). Both components are shown in Figure 2A.

Hill's equation of muscle contraction dynamics is given in a form that describes normalized muscle force $\hat{F}$_*v*_ = *F*_*v*_/*F*_*max*_ as a hyperbolic function of normalized (lengthening) velocity $\hat{v}$ = *v*/*v*_*max*_. In the case of contraction (*v* < 0):

(4) $${\hat{F}}_{v}(\hat{v})=\frac{1+\hat{v}}{1-\hat{v}/k_{sh}}$$

with *k*_*sh*_ regulating the curvature of the function (McMahon, 1984). For lengthening muscle (*v* > 0) an analog but inverted hyperbola is often used, which is parameterized by *k*_*max*_, *k*_*le*_, and *k*_*m*_, which describe respectively the asymptotic value lim_*v* → ∞_ $\hat{F}$_*v*_, the curvature, and the slope at *v* = 0 as a multiple of the corresponding slope in the case of contraction (Kistemaker et al., 2006). Such a hyperbola is given by

(5) $${\hat{F}}_{v}(\hat{v})=\frac{k_{l}-k_{max}\hat{v}}{k_{l}-\hat{v}}\text{, }k_{l}=\frac{k_{le}(1-k_{max})}{k_{m}(1+k_{le})}$$

The resulting function for concentric as well as eccentric contraction is shown in Figure 2B.

The total force *F* produced by a muscle depends on $\hat{F}$_*a*_, $\hat{F}$_*p*_, and $\hat{F}$_*v*_ in a multiplicative way (see Figure 2C):

(6) $$F=aF_{max}{\hat{F}}_{a}({\hat{l}}^{m}){\hat{F}}_{v}(\hat{v})+F_{max}{\hat{F}}_{p}({\hat{l}}^{m})$$

where *a* describes muscle activation dynamics and is implemented as a filter on neural excitation α, interpreted as firing rate in the range [0, 1], with different activation and deactivation rates β_*ac*_ and β_*de*_ respectively:

(7) $$\dot{a}=\{\begin{array}{ll} (\alpha -a)/\beta_{ac} & \text{if }\alpha \geq a,\\ (\alpha -a)/\beta_{de} & \text{if }\alpha <a \end{array}$$

Muscle length *l*^*m*^ is calculated from arm kinematics based on a geometric model of muscle paths wrapping around joint capsules as proposed by Houk et al. (2002). Given a muscle's points of origin and insertion (*p*_*o*, *i*_), the path depends only on the radii *r*_1,2_ of the spherical joint capsules it may wrap. The same model also determines each muscle's changing moment arm *m*_*a*_, from which we ultimately compute the torque η^*m*^ = *m*_*a*_*F* that is applied by a muscle at a joint.

Finally, summing the two individual muscle torques acting on the same joint we arrive at the total external torques η_*i*_ (*i* ∈ {1,2}). These are substituted in Equation 1, which allows us to rearrange for joint accelerations $\ddot{\theta }$_*i*_ and to integrate the dynamical equation using the Euler method (step size of 0.001).

Tendons were omitted from the model. The inclusion of tendons is important in some contexts, such as studies of the physiological mechanism underlying the disambiguation of musculotendon length (see e.g., Kistemaker et al., 2012), which we here take for granted. But the majority of muscles in the human upper arm feature short tendons, with ratios of tendon slack length to muscle fiber length on the order of 1–2 (Zajac, 1989; Garner and Pandy, 2003; Kistemaker et al., 2007), as opposed to long elastic tendons with ratios on the order of 10. Such tendons can store only small amounts of elastic energy and therefore have little effect on overall movement dynamics (Zajac, 1989; Gribble et al., 1998; Murray et al., 2000). At sub-maximal levels of muscle activation, as used here, their effect on static musculotendon properties (e.g., the force-length curve) is small too (Zajac, 1989). On that basis, and since current evidence does not suggest a special role for tendons in the creation or compensation of interaction torques, we consider the omission of (or assumption of an inelastic) tendon to be admissible for the purpose of our investigation.

All muscle parameters, such as maximum isometric forces or muscle excursion, were limited to ranges found in the human upper limb (Zajac, 1989; Lemay and Crago, 1996; Garner and Pandy, 2003), and are summarized in Table 1.

**Table 1:** **Summary of optimizable model parameters and their ranges, as well as fixed muscle parameters**.

**Parameter**	**Range**	**Description**
**MUSCLE**		
*p*_*o*, *i*_	[0.06, 0.26] m	Dist. of insertions from joint
*F*_*max*_	[100, 2000] N	Maximum isometric force
*l*_0_	[0.01, 1.5] *l*_*max*_	Optimum length
*v*_*max*_	[8, 12] *l*_0_/*s*	Max. contraction velocity
*r*_1,2_	[0.01, 0.05] m	Joint capsule radii
**NEURAL**		
τ	[0.01, 1] s	Time constant
θ	[−10, 10]	Bias
*w*	[−10, 10]	Connection weights
*k*	[0.1, 10]	Slope of transfer function
**THRESHOLD CONTROL**		
*k*_*p*_	[0, 6]	Position feedback gain
*k*_*v*_	[0, 0.5]	Velocity feedback gain
*k*_*d*_	[0, 1]	Damping gain
*p*_*v*, *d*_	[0.25, 2]	Viscosity exponents
λ^*co*^	[0, 0.3]	Open-loop cocontraction
**FIXED PARAMETERS**		
*k*_*sh*_, *k*_*le*_	0.25	Hill-function curvature
*k*_*max*_	1.5	Max. eccentric force
*k*_*m*_	2	Hill slope multiplier at *v* = 0
β_*ac*_	0.04 s	Muscle activation time scale
β_*de*_	0.07 s	Muscle deactivation time scale

See Materials and Methods for further detail.

### 2.3. Spinal model

We include in our neural model of spinal circuitry only the most well-known afferents, interneurons and connections. The architecture in its basic form is similar to previous models (Bullock and Grossberg, 1991; Lan et al., 2005; McCrea and Rybak, 2008; Raphael et al., 2010) and can be described as an antagonistically organized pattern generator (also see Pierrot-Deseilligny and Burke, 2005, for an overview of the connectivity). In particular, we include in our model the following interneurons and their connections (see Figure 1).

The Ia pathway includes monosynaptic excitation of the (homonymous) alpha motor neuron pool (α-MN) through muscle spindles, i.e., the myotatic (stretch) reflex; reciprocal inhibition of the antagonist α-MN via Ia interneurons (IaIn); and reciprocal inhibition between IaIns. IaIn further receive descending connections, which in this model carry signals related to the desired contraction of the corresponding muscle.

On the Ib pathway, inhibitory interneurons (IbIn) mediate autogenic inhibition of the homonymous α-MN via afferents from Golgi tendon organs, and also reciprocally inhibit each other. Optional in our model are the Ib reciprocal excitation of antagonist α-MN (interneurons omitted for simplicity), and Ia projections to IbIn (Jankowska, 1992). In addition, Ib afferents project to spinal circuits regulating adjacent joints and form connections there with IbIn and α-MN.

Modeled feedback from Ib afferents is based on the observation that the ensemble activity of Golgi tendon organs provides an estimate of the total force acting on a muscle over a large range of force production (Crago et al., 1982; Mileusnic and Loeb, 2009). Therefore, modeled Ib afferents here signal the (normalized) muscle force $\hat{F}$. The model also includes recurrent inhibition of homonymous α-MN via Renshaw cells, which reciprocally inhibit each other.

All interneurons in this circuit are modeled as leaky integrators with logistic transfer function, a model commonly used to describe the average neural firing rate as a function of stimulus (e.g., Dayan and Abbott, 2001, pp. 33, 57):

(8) $$\tau {\dot{y}}_{i}=-y_{i}+\sum_{j\;=\;1}^{n}w_{ji}\sigma (y_{j}+\theta_{j})$$

where *y*_*i*_ is the activation of neuron *i*, τ its time constant, *w*_*ji*_ the strength of the connection from neuron *j* to *i*, θ a bias term, and σ(*x*) = 1/(1 + *e*^−*kx*^) the logistic activation function with k specifying its steepness. All parameters describing this equation are subject to optimization.

### 2.4. Threshold control

The threshold control formulation of the EP theory, the λ-model, assumes that descending motor signals are integrated at the α-MN membrane with afferent feedback from muscles, such that changes in central commands shift the threshold at which muscles become active (Feldman and Levin, 1995). When a muscle is stretched, the resulting afferent influence will lead to an increase in membrane potential until the muscle reaches a length at which the threshold is exceeded and the motor neuron starts firing. The resulting activation produces muscle shortening and thus tends to move it closer to the threshold length, thereby establishing an equilibrium in spatial coordinates (muscle lengths).

Models of this kind are consistent with empirically observed levels of damping, stiffness, and feedback delays (St-Onge et al., 1997; Gribble et al., 1998; Kistemaker et al., 2006; Pilon and Feldman, 2006), and have successfully been employed to address problems such as motor redundancy (Balasubramaniam and Feldman, 2004), sense of effort (Feldman and Latash, 1982), the relation between kinematics, dynamics and EMG patterns in reaching movements (Feldman et al., 1990; Gribble et al., 1998), load adaptation (Gribble and Ostry, 2000) and anticipatory grip-force modulation (Pilon et al., 2007).

Few studies have addressed the problem of interaction torques in the context of the EP theory. In Flash and Gurevich (1997) the authors propose an EP model that addresses adaptation to loads (such as those arising internally), which requires for each new load the tuning of limb stiffness and the modification of the time-course of the EP shift based on knowledge of the load's force and joint stiffness. Gribble and Ostry (2000) have shown that a simple learning mechanism can make use of only the positional error resulting from unexpected loads to learn for each movement corrections of otherwise simple motor commands. Also, Flanagan et al. (1993) have used an EP model to investigate the nature of control signals underlying two-joint arm movements, but did not systematically vary the direction of movements in a way that would have allowed them to study the effect of interaction torques. Our model differs from these in that it aims to identify a single neuromuscular system accommodating interaction torques independently of their magnitude and direction, i.e., without a different set of compensatory motor signals for each possible movement.

We assume that muscle spindles (together with the complex of static and dynamic γ-MN) can provide information about both muscle length and velocity via type Ia and II afferents. This feedback is used in the model to measure deviations from muscle threshold length λ and acts so as to minimize it (directly via the stretch reflex and indirectly via input to the interneurons). Similar to other models of threshold control (Feldman et al., 1990; Kistemaker et al., 2006), a muscle's α-MN pool activity at time t integrates central commands and afferent feedback according to the following equation:

(9) $$\alpha_{t}={\left[k_{p}(l_{t\;-\;\delta }-\lambda_{t})+k_{v}{\left({\dot{l}}_{t\;-\;\delta }-{\dot{\lambda }}_{t}\right)}^{p_{v}}+k_{d}{\dot{l}}_{t\;-\;\delta }^{p_{d}}+N\right]}_{0}^{1}$$

(10) $$\lambda \;=\lambda^{d}-\lambda^{co}$$

Here *l* is muscle length, λ the commanded muscle threshold length, δ a feedback delay, *k*_*p*, *v*, *d*_ gain parameters controlling the effect of position error, velocity error and damping respectively, the function [*x*]^1^_0_ clamps its argument to the interval [0, 1], and N summarizes the influences of all spinal interneurons with connections to α-MNs. The reference velocity component has been proposed by McIntyre and Bizzi (1993) to account for fast movements executed with low stiffness, and the exponents *p*_*v*, *d*_ allow for modeling of non-linear viscosity effects (Gielen et al., 1984). Both these extensions of the original λ-model are optional, and we will show that they are unnecessary when spinal circuitry is taken into account, but that at least one is needed if the contribution of these circuits is omitted. The duration of the feedback delay is 0.025 s, based on the short-latency EMG response to unloading of human arm muscles (Houk and Rymer, 1981).

For multiple muscle systems, such as the antagonistic setup used here, threshold control proposes that the descending signal λ consists of two additive components, one of which shifts the position of the combined equilibrium of the system, while the other specifies a range of muscle coactivation around the equilibrium point (Feldman and Levin, 1995). These two components are denoted as λ^*d*^ and λ^*co*^ in the above equation. In principle, independence of the coactivation component from the positional component can be achieved through coordinative processes in spinal circuits (Feldman, 1993), or centrally using information about muscle and skeletal morphology (e.g., in the form of a learned mapping). For simplicity, we here let the optimization procedure select the coactivation level λ^*co*^.

For reasons of stability in the reciprocally organized spinal circuits, which in some configurations can be prone to undesired reverberations, all interneurons receive a movement-unspecific, descending, GO signal (Bullock et al., 1998; Raphael et al., 2010), which has the potential to gate the contribution of spinal interneurons to α-MN activity. The GO signal is set to 1 at the beginning of movement and gradually drops to 0 at the end:

(11) $$\begin{array}{l} \;GO(t_{0})=1\\ GO(t+\Delta t)=\{\begin{array}{ll} GO(t) & \text{if }t\leq T\\ 0.95GO(t) & \text{if }t>T \end{array} \end{array}$$

where *t*_0_ is the beginning and *T* the desired duration of the movement. Because of this time course, the GO signal cannot modulate neural dynamics during the execution of the movement, but can only alter interneuronal contributions after the movement terminates (and thus potentially prevent undesired oscillations when the limb is supposed to be at rest again).

### 2.5. Simulated movements and control signals

**Figure 3:**
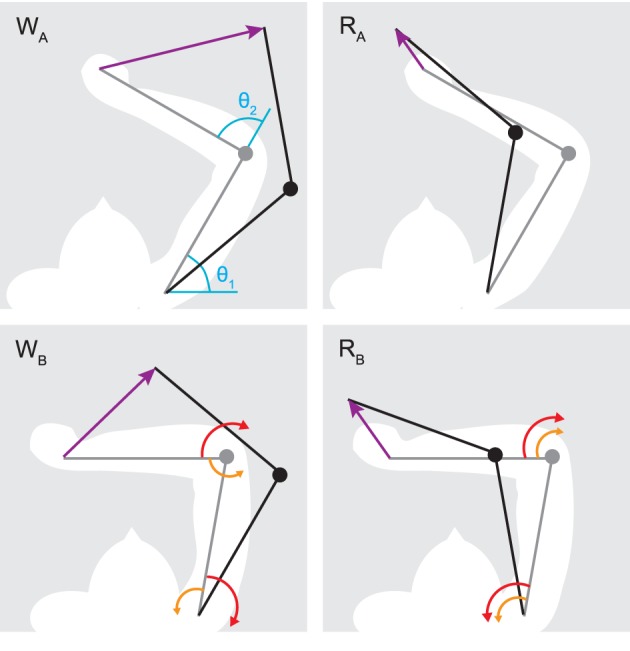
**Initial (gray) and target poses (black) for reaching (*R*_*A*,*B*_) and whipping movements (*W*_*A*,*B*_)**. Purple arrows indicate the desired hand path. In blue are shown the origins for shoulder (θ_1_) and elbow (θ_2_) joint angles. Red arrows indicate the direction of muscle torque required to initiate the desired motion about one joint, and orange arrows the direction of interaction torques due to motion about the other. Generally, muscle torques applied at one joint result in interaction torques of the opposite direction at the adjacent joint. This leads to interaction torques assisting the motion in the case of reaching movements, and resisting it during whipping movements. Note that all movements involve joint rotations of the same amplitude (±20° for the shoulder and −30° for the elbow), only their direction changes.

We consider two types of arm movements (Figure 3): “whipping” movements, where elbow and shoulder joints move in the same direction, and “reaching” movements, where the two joints move in opposite directions. Interaction torques broadly oppose the movement in the case of whipping and assist it in the case of reaching, at least in the initial phase (Gribble and Ostry, 1999; Galloway and Koshland, 2002). We try to identify spinal circuits that can produce smooth hand trajectories in both of these conditions. We therefore simulate four distinct movements. For each of two starting poses *S*_*A*_ and *S*_*B*_, specified in shoulder (θ_1_) and elbow (θ_2_) joint angle coordinates—*S*_*A*_:{θ_1_ = 60°, θ_2_ = 90°} and *S*_*B*_:{θ_1_ = 80°, θ_2_ = 110°}—we hold desired elbow kinematics constant (a 30° flexion) while changing the direction of shoulder movement (20° flexion or extension). This results in different directions of interaction torques arising at the elbow. A similar regime has been used in experiments with human participants (Gribble and Ostry, 1999). The duration of the desired movements is 0.3 s, which implies moderate average rotational velocities of 100°/s and 66.67°/s for the elbow and shoulder joint respectively. Note that as a result of the non-linear nature of arm kinematics, the four movements all differ in the distance traveled by the hand. These are *W*_*A*_ = 0.32, *R*_*A*_ = 0.11, *W*_*B*_ = 0.28, and *R*_*B*_ = 0.13 m, where *W* and *R* denote whipping and reaching movements with subscripts A and B indicating the starting position.

**Figure 4:**
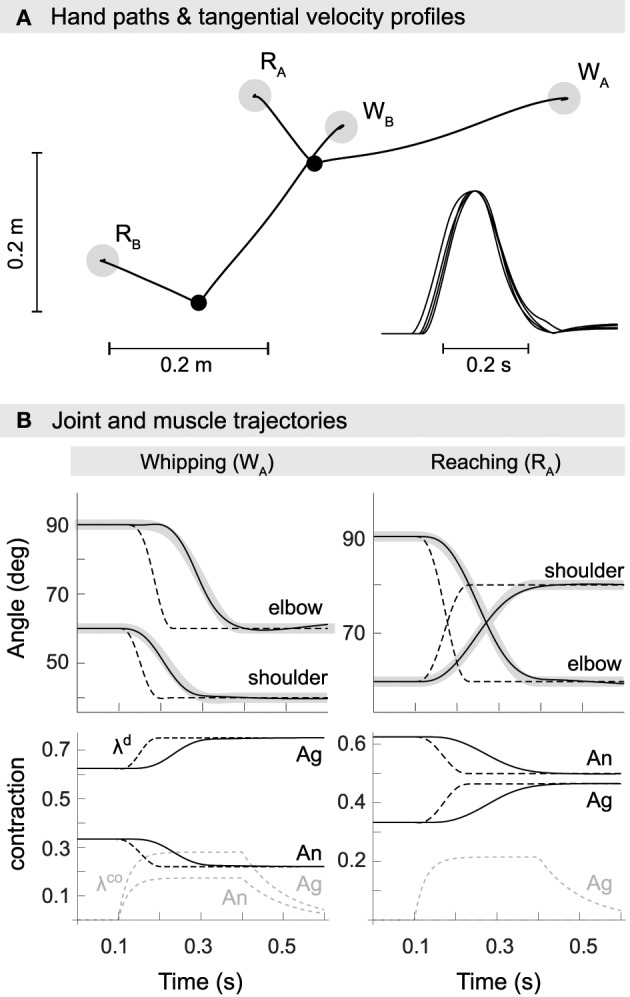
**Kinematics of model reaching and whipping movements**. **(A)** Hand paths in extrinsic space. Gray disks indicate target positions for reaching (*R*_*A*,*B*_) and whipping movements (*W*_*A*,*B*_). Black points indicate initial positions. Trajectories are approximately straight with small hooks at the target positions. Inset at the bottom right are normalized tangential velocity profiles scaled by maximum velocity and shifted such that peaks coincide. The profiles are approximately bell-shaped with some overshoot. **(B)** Trajectories in joint space (first row). In black are shown actual and in gray desired joint trajectories. Also plotted as dashed lines are the commanded joint trajectories, i.e., the virtual equilibrium joint angles corresponding to the central commands λ^*d*^. Second row: muscle trajectories for both agonist and antagonist of the shoulder. Muscle excursion is here measured as a proportion of maximum contraction, i.e., a value of 1 corresponds to a muscle being maximally contracted (at its shortest) and a value of 0 to it being minimally contracted (at its longest). Shown are the commanded muscle threshold λ^*d*^ (black, dashed), actual muscle contraction (black) and the open-loop component λ^*co*^ (gray, dashed).

The following scheme is used to derive central command signals λ^*d*^ for the four movements from their corresponding initial and target postures. Based on the assumption that hand trajectories are controlled in extrinsic space (Morasso, 1981; Flash and Hogan, 1985), the two postures, given in joint coordinates, are translated into hand positions (the tip of the forearm segment in the model) using forward kinematics. From initial and final hand positions we then derive a commanded trajectory, sometimes called the “virtual equilibrium trajectory,” which corresponds to the true equilibrium of the system only in the absence of load perturbations. Based on the finding in similar models that the equilibrium-point trajectory might lead the actual movement, i.e., reach the final position before the end of the movement (see e.g., Ghafouri and Feldman 2001), we allow the commanded trajectory to be shorter. Its duration as a fraction of the desired movement is an optimized parameter. To avoid discontinuities in the control signals, which could induce undesired oscillations, the commanded trajectory changes smoothly between the initial and final hand position. Finally, using inverse kinematics, at each time step the commanded hand position is transformed into commanded muscle threshold lengths λ^*d*^. In other words, each muscle's λ^*d*^ for a given commanded hand position is the length of that muscle at the corresponding position. The open-loop component λ^*co*^ gradually increases from 0 to its selected level, remains constant throughout the movement, and then gradually relaxes back to 0 (Feldman and Levin, 1995). Example time series of the two control signals are shown in Figure 4B.

In summary, the continuous time-varying control signals are fully determined by three parameters, namely the initial and final hand position as well as a coactivation level, and it is assumed that both the virtual equilibrium position of the hand as well as the open-loop component change smoothly. An inverse kinematic mapping is used to translate hand positions into muscle threshold lengths.

### 2.6. Optimization

We use a genetic algorithm (GA) to search for parameters of the spinal circuits and muscles such that the combined system produces in all movement conditions smooth hand motion as described by a minimum jerk trajectory. This is done in two separate stages.

#### 2.6.1. Muscle parameters

First, a set of minimally valid muscle parameters is identified that subsequently remains fixed (22 parameters in total, see Table 1). We employ only two criteria in the search for these muscle parameters. Firstly, the parameters have to fall within physiologically plausible ranges (also provided in Table 1). Secondly, the resulting musculoskeletal system, when driven with static and sub-maximal activations, has to exhibit stable equilibria at least over the joint range employed in subsequent simulations. This is a property too of biomechanically more realistic simulations and most probably also humans (Kistemaker et al., 2007). The overall complexity of the musculoskeletal model is kept at a level sufficient not to dissolve the problem of interaction torques in the first place, and is not meant to be a high-fidelity reproduction of the human upper arm complex. For this reason the set of lumped muscles was determined using the minimal criteria mentioned above, rather than an average over empirical measures, which would be inappropriate to map to the setup used here. Note that the search criteria for muscle parameters do not include the minimization of interaction torques or any other related objective. In fact, no online control is used at this stage at all, and spinal networks are completely ignored. This ensures that the identification of the muscle setup is completely independent of the subsequent problem of optimizing spinal feedback control.

#### 2.6.2. Spinal network parameters

Parameters related to neural circuits and control signals (133 in total, see Table 1) are optimized with the goal of producing (1) smooth hand motion, (2) low levels of co-activation and (3) insignificant muscle forces before and after movement. To this end we define an objective function that consists of the three components *f*_*D*_, *f*_*C*_, and *f*_*F*_, which capture each of the previous criteria respectively. The overall performance of the system for a single movement trial, and given a particular set of parameters, is then measured by simply multiplying the three individual components, which we describe next in detail.

As a criterion for smooth hand movements that reproduce empirically observed kinematic invariants we first define an ideal minimum-jerk trajectory (Flash and Hogan, 1985), $\vec{r}$(*t*), between the hand's initial and final position. The performance criterion *f*_*D*_ then depends on the mean squared error *D* between actual hand position $\vec{p}$ and reference $\vec{r}$ along the trajectory, at the relative time lag that minimizes the error between the two time series:

(12) $$D=\underset{-0.1\;<\;d\;<\;0.1}{\min}\sum_{t\;=\;0}^{T}\|{\vec{p}}_{t}-{\vec{r}}_{t-d}{\|}^{2}$$

(13) $$f_{D}=1-\sqrt{\frac{D}{T/dt}}$$

where *t* is time (in discrete steps of *dt* = 0.001 s) and T the movement duration. The latter includes periods without motion before and after the actual movement (0.1 s and 0.3 s respectively). *D* is the movement error for the best matching time lag *d*, and the final performance measure *f*_*D*_ scales the error such that its maximum is 1.

Though the periods of stationarity implicitly tend to suppress oscillations at the beginning and end of a movement, we found it necessary to further constrain solutions to reduce force production before and after the desired movement. To this end, we introduce a performance measure *f*_*F*_ which decreases in proportion to average muscle forces 〈$\hat{F}$〉 greater than 4% of their isometric maximum:

(14) $${\langle \hat{F}\rangle }_{i,t}\;=\;\frac{1}{2}{\langle {\hat{F}}^{i}(t_{0}+t)+{\hat{F}}^{i}(T-t)\rangle }_{i,t}$$

(15) $$f_{F}\;=\{\begin{array}{ll} 0.04/{\langle \hat{F}\rangle }_{i,t}, & \text{if }{\langle \hat{F}\rangle }_{i,t}>0.04\\ 1, & \text{if }{\langle \hat{F}\rangle }_{i,t}\leq 0.04 \end{array}$$

where the notation 〈*x*^*i*^〉_*i*, *t*_ refers to the average of variable x measured over all components *i* of the variable and over the duration of time given by *t*. Here, the component 〈$\hat{F}$〉_*i*, *t*_ measures the normalized muscle force averaged over the first and last 0.1 s of a trial (0 ≤ *t* ≤ 0.1 *s*) and over all muscles *i* = 1, …, 4 (*t*_0_ is the beginning of a trial and T its end). For average muscle forces 〈$\hat{F}$〉 greater than 0.04 (4% of maximum isometric force), the corresponding component *f*_*F*_ quickly decreases toward 0, and its maximum is 1 if average muscle forces remain below this threshold.

A final performance constraint *f*_*C*_ aims to rule out solutions that exhibit high levels of joint stiffness, which would allow muscle forces to dominate over the effect of interaction torques. It measure for each joint *j* a measure *C*_*j*_ which punishes muscle activation patterns in which coactivation is greater than a given threshold:

(16) $$C_{j}=1-\max\left(0,\underset{0\;\leq \;t\;\leq \;T}{\max}\left(\min(\alpha_{t}^{j_{f}},\alpha_{t}^{j_{e}})\right)-0.2\right)\;$$

(17) $$f_{C}=\prod C_{j}$$

Here, *C*_*j*_ becomes increasingly smaller than 1 when the coactivation of muscles acting at joint *j*, measured by the minimum of flexor and extensor α-MN activations α^*j*_*e*, *f*_^, becomes greater than 20% of the maximum at any point throughout the trial. The final performance component *f*_*C*_ is then calculated by multiplying the measures *C*_*j*_ obtained for different joints *j* to ensure that the constraint is observed by all joints.

Finally, the performance for trial *i* is given by *f*^*i*^ = *f*_*D*_ · *f*_*F*_ · *f*_*C*_, and the overall performance across all trials by the product *f* = ∏^*N*^_*i*_*f*^*i*^, with N being the number of trials (movements). Individual and composite performance measures are constructed such that their maximum is 1 in the case of optimal performance and 0 in the worst case. Using the product prevents optimization of a subset of movements at the expense of others.

All parameters are optimized by maximizing performance *f* using a version of the microbial genetic algorithm (Harvey, 2011), with mutation implemented as a random offset vector in the unit hypersphere (vector length chosen from a Gaussian distribution, and direction from a uniform distribution).

The set of model parameters optimized in this stage consists of synaptic strengths, neural biases and time constants, feedback gains, as well as the duration of the commanded equilibrium shift and the cocontraction signals λ^*co*^ (Table 1). Only the cocontraction components are optimized on a per-movement basis, i.e., a different set of λ^*co*^ is identified for each target position.

Note that the optimization procedure is not meant to be a model of the developmental phase establishing the appropriate connectivity of spinal networks in humans (though potential adaptive processes underlying it are mentioned in the discussion). In particular, we do not claim that the CNS uses a minimum jerk criterion to learn how to perform reaching movements, rather than minimizing, say, energy expenditure or variance in the presence of noise. The sole purpose of the optimization is to identify whether there exists at all a spinal circuit, given the constraints of realistic network topology and the right kind of proprioceptive stimuli, that allows for the feedback compensation of interaction torques.

## 3. Results

In this section we report on the best neuromuscular system identified in 21 independent runs of the optimization procedure (a list of all fixed and optimized parameters is provided in a supplementary file accompanying this text). This model achieves 98.97% of maximum performance. Both additional performance constraints (coactivation less than 20% and average muscle force less than 4% before and after movement) are completely satisfied, hence the performance level is due solely to trajectory error. Table 2 summarizes movement errors and kinematic indices for the four movements.

**Table 2:** **Summary of movement kinematics for reaching and whipping movements produced by optimized spinal circuit**.

	**MED [mm]**	***v***^***t***^_***max***_ **[m/s]**	***v***^***e***^_***max***_ **[deg/s]**	***v***^***s***^_***max***_ **[deg/s]**
*W*_*A*_	3.5	1.92	277	187
*R*_*A*_	1.9	0.73	194	122
*W*_*B*_	2.7	1.85	251	184
*R*_*B*_	1.4	0.82	185	125

MED is the mean Euclidean distance between actual and desired hand paths; *v*^*t*^_*max*_ the maximum tangential hand velocity; and *v*^*e*, *s*^_*max*_ elbow and shoulder maximum rotational velocities.

The mean Euclidean distance (MED) between actual and reference trajectory is on average 3.1 mm for whipping movements (which cover an average movement distance of 30 cm), and 1.6 mm for reaching movements (average movement distance of 12 cm). For comparison, when the model is optimized without contribution from spinal interneurons and movements are under sole control of the threshold model as described in equation (9), the MED for *W*_*A*_ is 25 mm and the average across all four movements 11.8 mm (see below section on the role of IbIn for a more detailed comparison). The results show that the optimization procedure successfully identifies spinal circuits that produce minimum jerk-like hand trajectories.

### 3.1. Morphology

A summary of the lumped muscle setup is shown in Table 3. Note that although muscles were constrained to be symmetric, excursion varies between flexors and extensors in the model (because the range of motion is asymmetric). Also, like mono-articular human elbow muscles, extensors have a constant moment arm, while for flexors the moment arm is changing with joint angle. A salient feature of the optimized morphology, specifically muscle insertions and optimal lengths *l*_0_, is the fact that the excursion of all muscles is confined mostly to the ascending leg of the force-length curve, i.e., muscle lengths over the whole joint range are mostly smaller than their respective optimum length (where force production peaks). This can also be observed for the majority of human upper arm muscles (Murray et al., 2000; Garner and Pandy, 2003). Though it is not clear whether this is the reason, it increases the probability that the isometric moment-angle relationship of a joint (given by the sum of the isometric moment-angle curves of all muscles acting at the joint) exhibits a single stable equilibrium only (see e.g., Kistemaker et al., 2007).

**Table 3:** **Summary of muscle setup after optimization with static control signals for achieving stable equilibria at all positions required in subsequent feedback control**.

**Muscle**	**Origin [m]**	**Insertion [m]**	***l***^***m***^ **[*****m*****] Min**	**Optimal (*l***_**0**_**)**	**Max**	***F***_***max***_ **[N]**	***v***_***max***_ **[*****l***_***0***_/***s*****]**
Elbow flexor	0.239	0.078	0.168	0.31	0.336	138	11.6
Elbow extensor	0.239	0.078	0.336	0.447	0.468	138	11.6
Shoulder flexor	0.254	0.06	0.198	0.359	0.334	1523	11.2
Shoulder extensor	0.254	0.06	0.334	0.477	0.455	1523	11.2

Origin and insertion points are measured as distances from the joint axis.

The maximum isometric force *F*_*max*_ for elbow muscles was constrained to be smaller than that of the shoulder muscles, since the latter need to support and transport a larger mass than the former. Also, in the literature the strongest shoulder muscles (such as the deltoid) are consistently reported to be stronger than elbow muscles (Nijhof and Kouwenhoven, 2000; Garner and Pandy, 2003; Holzbaur et al., 2005). The strength difference in our optimized model is rather big. For the purpose of our investigation, however, this would pose a problem only if optimized muscles were unrealistically strong, and if neural control would exploit this strength to overpower the interaction torques at the elbow joint. But this is not the case. As we demonstrate below, the combination of muscle strength and activation levels leads to a realistic range of dynamic torques throughout the movement. For example, interaction torques can reach the same level as muscle torques, and maximum shoulder torques (on the order of 10 Nm), are a multiple of maximum elbow torques (about 2 Nm), similar to measures from human subjects (e.g., Galloway and Koshland, 2002).

Finally, maximum contraction velocities (*v*_*max*_ approx. 11.4 *l*_0_/*s*) fall within a physiologically plausible range. Zajac (1989), for example, assumes an average of about 10 *l*_0_/*s*; Ranatunga (1984) measured values between 7 and 13 *l*_0_/*s*; for the empirically based model used in Kistemaker et al. (2007) *v*_*max*_ is not specified numerically, but visual inspection indicates values about or greater than 10.

The described morphology is only one of a wide range discovered by the search procedure. Others were found with similar performance but great variation in selected parameter values, indicating that the task underspecifies the required musculoskeletal properties.

### 3.2. Movement kinematics

To assess whether the optimized model reproduces empirically observed kinematic invariants (Morasso, 1981; Soechting and Lacquaniti, 1981; Atkeson and Hollerbach, 1985; Flash, 1987; Flanagan et al., 1993), in Figure 4 we show movements performed by the best optimized spinal circuit. Trajectories are approximately straight with slight curvature, feature small hooks at the target positions, and exhibit the characteristic bell-shaped velocity profile. This is true for all four movements, i.e., independent of the direction of interaction torques or starting posture.

In panel B, we plot example joint trajectories for movements *W*_*A*_ and *R*_*A*_. Consistent with earlier findings (Ghafouri and Feldman, 2001), we observe that the commanded trajectory (dashed) is significantly shorter than the movement period. The optimized duration covers 44.6% of the actual movement. Also note that the commanded (and desired) trajectories of individual joints are offset slightly in time, a result of their derivation through inverse kinematics from a hand path planned in extrinsic space.

### 3.3. Movement dynamics

**Figure 5:**
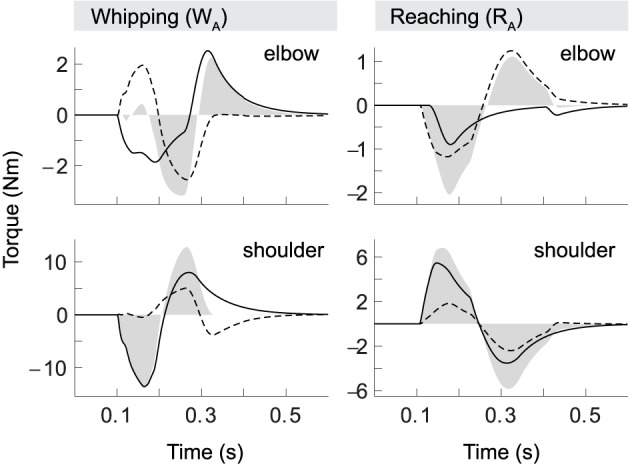
**Torque patterns for model whipping and reaching movements (*W*_*A*_ and *R*_*A*_)**. Elbow and shoulder torques are separated into muscle (black line), interaction (dashed) and net torques (filled).

The demonstrated kinematic invariants alone are not sufficient to imply active compensation, or accommodation, of interaction torques by the spinal cord. Although we know that central commands in our model carry no anticipative corrective components—the threshold shift is always of the same monotonic form—we need to show that the spinal cord can transform these identical control signals into muscle activation patterns that differ qualitatively with the direction of interaction torques (Cooke and Virji-Babul, 1995; Latash et al., 1995; Gribble and Ostry, 1999; Galloway and Koshland, 2002; Debicki and Gribble, 2005). Figure 5 shows torque patterns produced by our model for movements starting from initial posture *S*_*A*_ (corresponding to kinematics shown in Figure 4B).

Firstly, we observe that the interaction torque experienced in one joint (dashed) strongly correlates with the total torque (filled) in the other. Secondly, comparing whipping and reaching movements, we find that elbow torques vary with the direction of shoulder motion even though elbow kinematics are held constant. In whipping movements interaction torques due to shoulder movement initially oppose the movement of the elbow. This effect is significant, as peak interaction torque in the elbow is slightly greater than the muscle torque. Interestingly, in this case the two torque components almost cancel out, which leads to a delay in the onset of elbow motion of exactly the duration necessary to follow the desired hand trajectory. Whipping movement *W*_*B*_ (not shown) differs in this respect, in that the interaction torque here is slightly smaller than muscle torque, resulting in no such onset delay (again, as the desired hand movement requires). The shoulder, in contrast, is subject to only minimal interaction torques, and its movement is consequently dominated by muscle torques. This was also found to be the case for the second starting posture.

During reaching movements, in comparison, our model shows that interaction torques at the elbow initially assist the motion. They are equal in sign to the muscle torques and on the same order of magnitude. Though this effect is stronger in the elbow, interaction torques also assist shoulder motion, but in this case are significantly smaller than muscle torques. We thus find that both types of movement are characterized by a shoulder-centered pattern, in which initial shoulder motion is generally dominated by muscle torques, while elbow motion is produced with significant contribution from interaction torques.

The pattern of opposing and assisting effects of interaction torques also determines the overall muscle effort required. In the opposing case, elbow muscle torques need to be larger than in the assisting case to compensate for interaction torques, even though the kinematics of the motion is essentially the same (a 30° flexion) in both cases. This is particularly salient in elbow torques during reaching. Here the muscles do not contribute at all to the braking forces that terminate the movement, i.e., we observe no forces resulting from antagonist activity. The braking pulse in this case is exclusively due to interaction torques created by shoulder motion.

In short, the movement kinetics in our model confirm that the spinal circuitry successfully transforms simple descending control signals into muscle force patterns that qualitatively differ with the direction of interaction torques in such a manner as to accommodate them.

### 3.4. Neural dynamics

**Figure 6:**
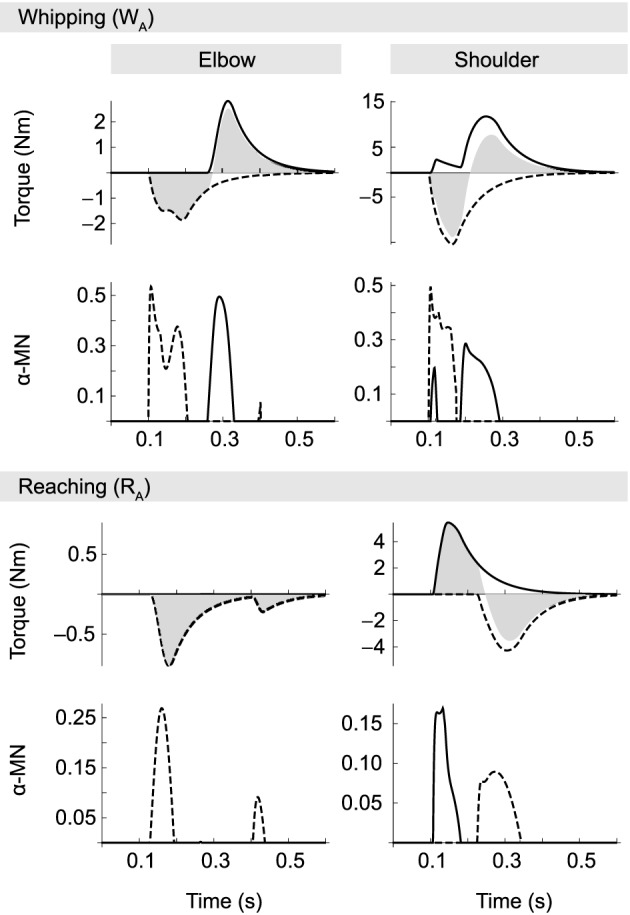
**α-MN activity (on the output scale of [0, 1]) and corresponding muscle torques for movements *W*_*A*_ and *R*_*A*_**. Solid traces correspond to the flexor and dashed lines to the extensor muscle in each joint. Filled gray areas indicate net muscle torques (i.e., the sum of flexor and extensor). In whipping movements, where interaction torques oppose the motion, muscle activity is generally larger than in reaching movements, even though in both cases absolute joint excursions are the same. During reaching movements muscle activity is smaller. Note in particular the absence of a braking pulse in the elbow. Elbow deceleration in this case is almost exclusively due to interactions torques produced by the shoulder.

In Figure 6 we plot the activity of α-MNs and the individual muscle torques they provoke (taking into account muscle activation dynamics and changing moment arms) for the same movements as shown in Figures 4, 5 (*W*_*A*_ and *R*_*A*_). Neural activity exhibits the characteristic bi- or tri-phasic burst patterns observed empirically (Ghez and Gordon, 1987), i.e., we can generally identify an accelerating agonist burst followed by a decelerating antagonist burst, and sometimes a third agonist burst arresting the motion.

Not surprisingly, given the torque patterns described above, elbow muscle activity varies with the direction of motion in the shoulder, despite virtually identical elbow kinematics. Muscle activity is generally greater when both joints move in the same direction, i.e., when interaction torques oppose the movement (whipping). For example, integrating over the corresponding area under the curve, we find that motor neuron activity associated with the first elbow extensor burst (which is the agonist in these movements) is approximately 0.033 for the whipping movement, but three times smaller (0.011) for the reaching movement. The same is true for the antagonist (here the elbow flexor). In the former case the area of its burst is 0.024, and when interaction torques assist the motion it vanishes completely.

In contrast with some empirical data, we observe in our model no systematic time lag between onsets of activity in agonists acting on different joints; in particular, we find no temporal organization from proximal to distal joints (Karst and Hasan, 1991; Gribble and Ostry, 1999). Even though such a lag seems to be present in movement *R*_*A*_ (in Figure 6 compare first α-MN bursts of elbow and shoulder in the reaching condition), this was not the case for all reaching movements.

For reasons of space we do not present a full analysis of the neural dynamics exhibited by the optimized spinal circuitry. We suggest, however, an explanation for the suppression of the elbow antagonist burst during reaching movements. In the optimized spinal circuit, the antagonist burst (and its suppression) can be traced back to two opposing influences on its α-MN pool. First, spindle feedback excites the α-MN in proportion to deviations from desired position and velocity. But secondly, spindle activity also drives IaIn activity, and via connections from IaIn to homonymous IbIn (Jankowska, 1992) also the latter interneurons, which inhibit α-MN activity. A second inhibiting influence in the optimized network originates in IbIn intersegmental connections from muscles acting on the other joint. The size and shape of the antagonist burst therefore is the result of a balance between spindle feedback and IbIn activity, which in turn is modulated by Ia interneurons. In whipping movements, presumably because interaction torques initially oppose the movement, position and velocity errors initially grow relatively large, resulting in spindle feedback sufficient to overcome the aforementioned inhibiting factors. In reaching movements, on the other hand, interaction torques partially “do the work,” which leads to smaller deviations from the desired state, and hence spindle feedback that is more easily suppressed by the same inhibiting factors.

### 3.5. Generalization

**Figure 7:**
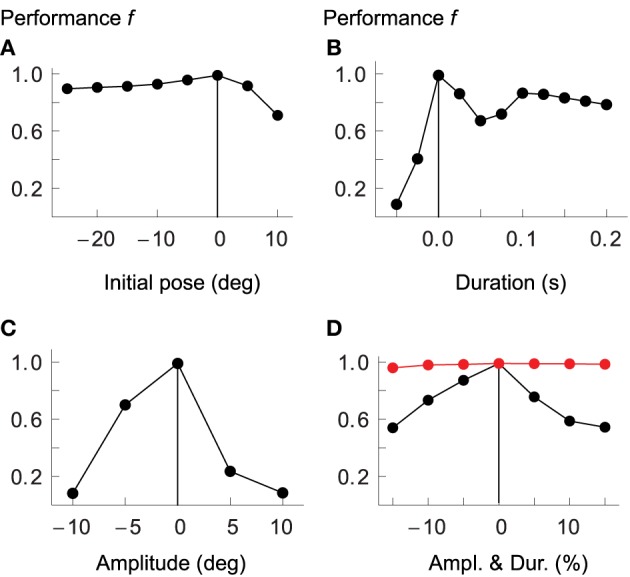
**Model generalization ability**. Performance of the optimized controller as the two starting postures are offset by a given number of degrees in joint space **(A)**; as movements vary in duration **(B)** or amplitude **(C)**; and as duration and amplitude are changed in proportion such as to maintain average velocity **(D)**. Measures on horizontal axes are relative to values used for optimization, which are indicated by vertical lines. Performance drops the more movements differ from the optimized kinematics. In red we plot performance when cocontraction signals are adapted separately for each desired movement.

The model presented above has been optimized for movements of a certain amplitude and speed (in joint space) and in two separate regions of the arm's workspace. Here we briefly address whether it generalizes to other types of movement that it has not been optimized for. We start by testing the model's performance as we shift the two starting postures in joint space by values ranging from −25° to +10° (elbow and shoulder angles are shifted by the same amount), while keeping duration and amplitude fixed at the original values. As Figure 7A demonstrates, the optimized controller shows specificity for the area of the workspace encountered during optimization. Performance decreases in both directions from the original postures.

Similarly, performance drops quickly as we change the duration of the movements to be shorter or longer than the original (panel B), or as the amplitudes are increased or decreased relative to those used during optimization (panel C). If we scale amplitude and duration equally, such that the average velocity remains constant, the drop in performance is less dramatic, but the overall picture is the same (panel D).

In the above tests, none of the system's control signals were re-adjusted for the varying movement kinematics. It cannot reasonably be expected, however, that the resulting movements should be well executed if, for example, the amount of cocontraction (the component λ^*co*^) is not tuned to the speed demands of the desired movement. It has been shown in human subjects, for example, that movement velocity correlates with muscle cocontraction (Gribble et al., 1998). Also, the balance of open-loop antagonist muscle activity specifies the equilibrium of the system in statics, implying that a wrong selection of this balance (given a specific target) could lead to spinal circuits and musculotendon system driving toward incompatible equilibria. At least these components of the feed-forward command therefore have to be chosen selectively for each particular movement. To test whether this is indeed sufficient to achieve reasonable performance, we first re-optimized all parameters of the original model after adding two new movements to the performance evaluation: the first is identical to *W*_*A*_, except that its duration is 0.4 s instead of 0.3 s; the second one is also similar to *W*_*A*_, but here both the duration and amplitude are 20% larger, such that the average velocity remains the same. The purpose of these additional evaluations is to avoid optimization of controllers that are overly specialized on the speed and amplitude demands of the four original movements. In a next step we then choose a few movements that the original system performs badly on and re-optimize only the cocontraction signals.

In Figure 7D we compare performance over a range of movement amplitudes (but constant average velocity) when the cocontraction signals are re-optimized (red) with those not adapted for each specific movement (black). In the former case performance remains almost constant, dropping no more than approximately 2% (from 98.9 to 97%). The performance of the non-adapted system, in contrast, drops to only 54%. We also tested a few of the other conditions presented in Figure 7. For example, the performance when desired joint rotations are reduced by 10% (while keeping the original duration fixed, thus leading to reduced average velocity), is 98.3%, compared to only 8% in the non-adapted system (see panel C). Equally, if the desired duration of the movement is increased by 20% while keeping the amplitude the same, performance of the adapted system is 98.7%, compared to only 67% for the non-adapted system. Performance is improved further if in addition to cocontraction signals we also tune spindle sensitivities to the desired movement, i.e., when we adapt the properties of the gamma pathways such that the strength of position and velocity feedback depends on the desired amplitude or velocity of the movement (data not shown).

**Figure 8:**
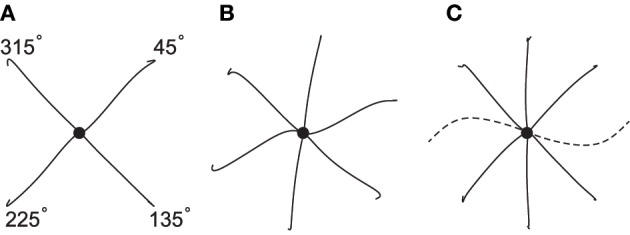
**Hand paths generated by a single spinal circuit when optimized for an increasing number of movement directions**. All movements have an amplitude of 10 cm, a duration of 0.3 s and proceed from the center (black dot) to targets spaced equally along a circle. Hand paths for most movements are approximately straight, except for those in the directions of 90° and 270° (dashed), which reflect a limitation of the biomechanical model.

To test the model's capacity for producing movements not only of different amplitudes and durations, but also in different directions, in Figure 8 we show the performance of the spinal circuit when optimized for an increasing number of movement directions. All movements here have a duration of 0.3 s and follow desired center-out hand paths 10 cm in length. Generally, hand paths are essentially straight (panel A, MED averaged over four movements: 1.8 mm). When the number of directions increases (panels B and C), some paths remain almost perfectly so, while others begin to show slight curvature (average MED for six movements: 2.3 mm; for eight movements: 4.2 mm). Almost all paths resemble the variation of curvatures observed in human reaching movements, except for two movements in panel C (toward targets located in the directions of 90° and 270°). Their large curvature and inaccurate termination reflect biomechanical constraints due to our muscle model, as these movements could not be optimized successfully even in isolation (average MED without these movements: 1.8 mm). This is indicative of the limitations of our simplified musculoskeletal system.

We also note that all of the optimizations described in this section were performed without the velocity error term in the threshold model, and without the power transformation of the viscosity term. Further tests, which we do no present here in detail, show that a control model without spinal interneurons depends on the presence of at least one of these terms to approach the performance of the spinal circuit.

In summary, when control parameters are tuned to the kinematics of the desired movement (e.g., cocontraction is matched to desired velocity), smooth movements can be performed accommodating interaction torques. This is the case for movements of different amplitude and velocity, in different areas of the workspace, and in different directions.

### 3.6. The role of ibin activity

As mentioned in the introduction, there is reason to speculate that intersegmental force feedback may provide a mechanism by which the nervous system compensates for interaction torques during multijoint movements. One can in fact conceive of three different roles: force-related feedback could modulate descending commands by acting in long loops through the CNS; coordinate muscles acting on different joints through intersegmental neural connections; or act indirectly through the mechanical coupling of joints and the sensed effect of internal loads in a manner akin to non-neural coordination mechanisms in the stick insect (Schmitz and Stein, 2000; Cruse et al., 2007).

**Figure 9:**
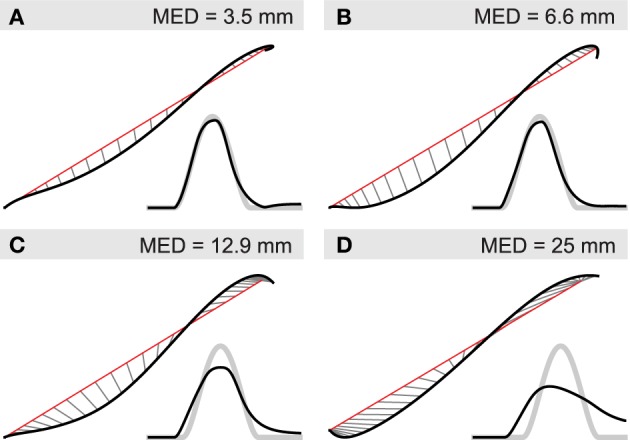
**Contribution of IbIn to movement kinematics**. Black lines indicate actual and red lines the desired hand position. Thin gray lines connect positions at equal points in time. Smaller insets show the corresponding velocity profile (black) and the desired velocity (thick gray). Deviations from straight line (minimum jerk) trajectories are indicated by mean Euclidean distance (MED). **(A)** Hand trajectory of unsevered spinal network for movement *W*_*A*_; **(B)** re-optimized system after removal of Ib connections between circuits regulating different joints; **(C)** re-optimized system without Ib interneurons; **(D)** system without interneurons.

We investigate here the latter two options by performing simulated ablation experiments in which we re-optimize the system's parameters after removal of different parts of the spinal circuit. Figure 9 shows hand trajectories for the worst of the four movements in each ablation condition, which happens to be *W*_*A*_ in all cases. Optimizing the system without intrasegmental Ib connections reduces the system's performance to a relatively small degree (compare panel A and B). The curvature of the trajectory becomes more pronounced and the movement is arrested less effectively. Movement error, measured by MED, increases from approximately 3.5 to 6.6 mm (and from 2.37 to 3.14 mm when averaged over all four movements). When Ib interneurons are completely removed from the network (panel C), curvature and endpoint oscillations become even more salient (MED = 12.9 mm; average MED = 6.24 mm). In addition, the time course of the trajectory deviates significantly from the reference. It initially leads the desired movement, but then fails to reach the required velocity and falls behind toward the end. For comparison we show in panel D the result when all interneurons are removed from the network, leaving only the threshold model, i.e., α-MNs and direct proprioceptive feedback. Although the trajectory in space shows no more curvature than seen in the other conditions, its time profile deviates much more strongly from the reference, which is most salient in the velocity profile.

### 3.7. Model sensitivity

**Figure 10:**
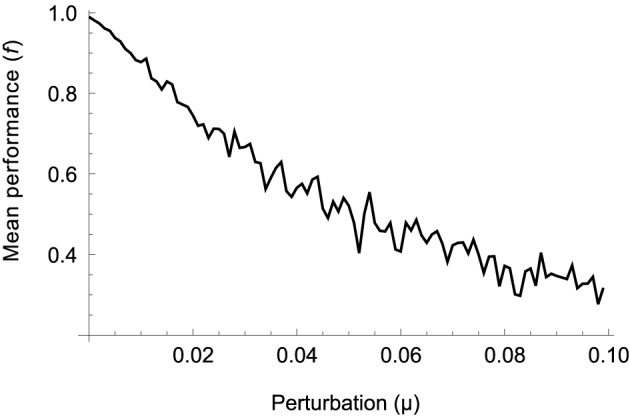
**Mean performance of 100 parameter vectors randomly sampled from a hypercube of size μ centered on the optimized solution**. Performance gradually drops as random solutions are located further away, on average, from the optimized one.

To assess the sensitivity of the model we evaluate its performance over a range of deviations from the solution optimized for the four original movements (*W*_*A*,*B*_ and *R*_*A*,*B*_). The optimization procedure encodes all parameters in a vector with component values in the range [0, 1]. For a given level of deviation μ we add random perturbations chosen uniformly from the range [−μ, μ] to all components, i.e., we select a random vector from within a hypercube of size μ centered on the optimized parameter vector. For every level of deviation we sample 100 such vectors and measure their mean performance. The result is shown in Figure 10.

It can be seen that performance drops gradually as the amount of deviation increases, indicating that the procedure has not found a “needle in a haystack.” The relative smoothness of the error surface suggests that other optimization methods, for example those based on trial-and-error or gradient descent, should also be able to find good solutions. Gradient-based learning using trajectory errors has in fact been demonstrated in a similar model of movement control using spinal-like neural networks (Raphael et al., 2010; Tsianos et al., 2011). On the other hand, performance starts decreasing immediately. The absence of a plateau around the optimal set of parameters suggests that there is not a great variety of different models leading to the same performance. However, since we are only probing (an increasing) neighborhood of one optimal solution, we cannot rule out that other solutions with similar performance exist in other regions of parameter space (in which case local optima might in fact hinder the performance of gradient-based algorithms).

## 4. Discussion

The dominating view in motor control today suggests that the CNS controls the body using intricate internal models of its kinematics and dynamics in order to predict and directly control the muscle forces required to perform a desired movement. An alternative view, expressed in the equilibrium point hypothesis, proposes that the combined dynamics of spinal circuitry and musculoskeletal system provide a level of abstraction in the control hierarchy that allows the CNS to plan and control movements without requiring a representation of complex bodily dynamics. In this paper we provide evidence that this is plausible. Instead of anticipating upcoming interaction torques and adjusting central control signals accordingly, our model suggests that the CNS may in some cases—such as the type of reaching movements considered here—be ignorant of musculoskeletal dynamics and offload the coordination of muscle forces required for a particular, kinematically defined, movement goal to circuitry at the spinal level. We do not rule out that prediction or implicit anticipatory mechanisms might be involved in other cases, such as faster or more complex movements.

Several studies (e.g., Almeida et al., 1995; Gottlieb et al., 1996; Gribble and Ostry, 1999) have found that during human limb movements EMG activity in muscles acting on one joint correlates with interaction torques arising from motion in another, and is often timed such that it precedes the onset of movement. These findings have been taken to imply that central motor commands are adjusted predictively to compensate for interaction torques. But it need not be true that any such adjustment takes place on the level of central control signals, nor that any form of prediction is involved. Firstly, it is clear that EMG activity has to vary systematically with upcoming interaction torques. If it were not to we would not observe hand paths that are approximately straight. Also, some muscle activity has to precede the movement, as it is necessary to initiate it. Furthermore, since we do not yet have sufficient knowledge about the precise nature of descending control signals, which only after integration with afferent and interneuronal signals results in observed EMG activity, it is impossible to conclusively deduce from current empirical data whether central commands are already adjusted for interaction torques, or whether they are transformed at a lower level to this effect. The model presented here demonstrates that accommodation of intersegmental loads on the spinal level is possible. Also, the fact that the temporal order of muscle activity—including prior to movement onset—seems to be relatively fixed and organized such that agonists in proximal joints precede distal ones, may reflect a more general organization of the control hierarchy, rather than specific and detailed predictions of upcoming dynamics.

The muscle and reflex model developed here involves significant simplifications when compared with the problem of controlling multijoint arm movements in the human body. For example, it does not take into account the effect of biarticular muscles, tendons or gravity. Spinal interneurons are modeled as simple leaky integrators, and fusimotor drives are represented only implicitly through the λ-model. Also, the Hill-type muscle model is employed here in its simplest form, ignoring, for example, the effect of calcium sensitivity on the force-length characteristic (Kistemaker et al., 2007), or the dependence of maximum shortening velocity on activation level (Chow and Darling, 1999). While the inclusion of tendons may be important, for example, when studying the physiological mechanism underlying the estimation of joint position (Kistemaker et al., 2012), we have argued in Section 2.2 that its effect on upper arm dynamics is limited. And if biarticular muscles are particularly well suited for internal load compensation (Gritsenko et al., 2011), then their inclusion in a model such as the one presented here should be expected to make the problem of feedback compensation easier. We also note that the problem of interaction torques in multi-segment limbs is in principal independent of how the joints are actuated, as long as the mechanism of actuation is not infinitely stiff (the problem hence also arises, for example, in any type of compliantly actuated robots). We therefore believe that none of the simplifications introduced in the model interfere with the basic goal of this study, which is to demonstrate that a single spinal-like neural network can transform simple descending control signals into muscle activation patterns that differ qualitatively with the direction and magnitude of interaction torques in a manner that is appropriate for the generation of smooth and straight hand trajectories. Moreover, the model achieves this with muscle and interaction torque patterns comparable to those observed empirically, and without the need for inverse dynamics calculations, prediction of upcoming loads, or having to learn adjustments to control signals for each individual movement. Nevertheless, we believe that further work aimed at testing the proposed mechanism in more realistic models is required to show how and whether the control scheme illustrated here is in fact employed in the case of human reaching movements and to generate detailed predictions that can be tested empirically and that go beyond the demonstration of a functional role for proprioception in intersegmental coordination (Ghez and Sainburg, 1995; Sainburg et al., 1995).

Despite the simplifications just mentioned, the model presents a significant complexity. This is mostly due to the need to represent spinal circuits explicitly in order to do justice to the hypothesis being investigated. Though the modeled circuits are still far simpler than real spinal circuits, we do not claim to have found the simplest or most efficient model able of interaction torque compensation, which was not our objective. In any case, the work presented here also demonstrates that the chosen methodology is indeed practical for asking questions about the potential functionality of spinal circuits, despite their complexity.

In addition to demonstrating the feasibility of feedback compensation for interaction torques in general, our model reproduces several features of reaching movements performed by humans. As demonstrated in Section 3.2, the hand trajectories generated are approximately straight and exhibit bell-shaped velocity profiles (Morasso, 1981; Flash, 1987; Flanagan et al., 1993). This is not surprising, since the movements were optimized to match minimium jerk profiles (Flash and Hogan, 1985). Exceptions from this mathematical ideal, such as uni- or bimodal curvature and hooks at the endpoints, are also in correspondence with empirical data (Flash and Hogan, 1985).

Regarding muscle and interaction torque patterns, Gribble and Ostry (1999) report that muscle torque applied at the elbow varies with the direction of shoulder motion across movements in which elbow kinematics are held constant (and, conversely, shoulder muscle activity varies with the direction of elbow motion when shoulder kinematics are constant). These dependencies are found even when active movement in only one joint is required (but the other is free to move) or when one of the joints is fixed (Debicki and Gribble, 2005). The direction of this covariation depends on whether the two joints move in the same or opposite direction. When shoulder and elbow move in the same direction, interaction torques arise in each joint that initially oppose that joint's intended motion and muscle torques in each joint increase with the emerging interaction torque. In contrast, when the joints move in opposite directions, interaction torques initially assist the desired movement, and muscle torques decrease proportionally. As we have shown in Section 3.3, our model behaves in the same way. Simulated elbow torques vary with shoulder kinematics, and overall effort depends on whether interaction torques are assistive or opposing. As reported in Section 3.4, this effect can be strong enough to completely suppress the normal antagonist burst in the elbow. In this case it is the interaction torque at the elbow alone that arrests the motion. Similar effects can be observed in human subjects, where in some cases assisting interaction torques reach levels such that an initial counteracting antagonist burst is required, instead of the more usual movement-initiating agonist activity (Cooke and Virji-Babul, 1995). Our model also exhibits a shoulder-centered pattern, where elbow dynamics are the result of approximately equal muscle and interaction torque contributions, while in the shoulder muscle torques are generally greater than the passively arising loads (at least initially). This is consistent with some empirical data (e.g., Galloway and Koshland 2002), although it has not been reported by Gribble and Ostry (1999).

With regard to the timing of muscle activity, we find no systematic temporal organization of agonist onsets from proximal to distal joints (Karst and Hasan, 1991; Gribble and Ostry, 1999). Investigation of a larger range of movements would be necessary to identify whether the model does or could exhibit such a strategy. We do, however, observe instances of onset delays in joint rotations (Karst and Hasan, 1991; Virji-Babul and Cooke, 1995), which result from the interaction of muscle forces and internal loads, and from the planning of movements in extrinsic space.

The optimized spinal circuits produce desired hand trajectories over a range of different amplitudes, speeds and directions (Section 3.5), with the exception of movements along a single direction, which was shown to be a limitation of the biomechanical model rather than its control. Further work would be required to determine whether the addition of biarticular muscles, for example, would broaden the operating range of the system, or whether the optimization procedure simply failed to identify a more capable biomechanical setup given the model's constraints. For the model to achieve good performance in all movement conditions, we have shown that some control parameters (such as coactivation level, or muscle spindle sensitivity) need to be selected on the basis of the desired movement speed and amplitude. But crucially, no details about the dynamics of the movement need to be known. The control signals are always simple—a monotonic shift in muscle reflex threshold and a constant level of coactivation throughout the movement—and do not depend on anticipated interaction torques or other aspects of the system's dynamics. The results suggest some further questions, however. For example, what are the *minimal* changes in control signals that allow for the control of movement speed or amplitude? Can the dynamics of the spinal circuits be adjusted using non-specific central control signals according to a simple scaling rule? Or does the CNS have to learn an explicit mapping between desired kinematics and control parameters?

We have speculated in the introduction that it might be intersegmental force feedback carried by Ib afferents that provides the mechanism by which the nervous system compensates for interaction torques. Such signals reliably encode muscle force (Mileusnic and Loeb, 2009), can be adjusted in sensitivity through interaction with Ia afferents (McCrea, 1992), and result in widespread modulation of motor neurons innervating muscles acting at adjacent joints (Jankowska et al., 1981). Moreover, functionally deafferented patients in some cases make systematic movement errors indicative of a failure to counteract interaction forces, which demonstrates a functional role for proprioception in the compensation of internal loads (Ghez and Sainburg, 1995; Sainburg et al., 1995). And motion-dependent feedback across spinal segments has been shown to modulate ongoing limb dynamics in the cat (Smith and Zernicke, 1987; Koshland and Smith, 1989). Our model only allows us to confirm that force feedback can indeed play a role in the compensation of internal loads, though we have not identified precisely what that role is. It is clear from our results that without the contribution of Ib afferents the performance of the model is greatly diminished. But both intra- as well as intersegmental effects of Ib afferents seem to contribute to the appropriate modulation of spinal neurodynamics. Further work is required to separate and study these two effects in more detail.

Our model does not address the question of how spinal circuitry might come to be organized in the manner presented here. The evolutionary optimization procedure does not serve as a model of how the appropriate connectivity could be learned. Nevertheless, it is known that neural circuits are subject to activity-dependent plasticity both in the developing as well as the mature spinal cord (Changeux and Danchin, 1976; Nelson et al., 1990; Lo and Poo, 1991; Wolpaw and Carp, 1993; Schouenborg, 2003; Wolpaw, 2010; Tahayori and Koceja, 2012). Moreover, the reciprocal structure typical of spinal circuits can arise through self-organization enabled by simple Hebbian-like learning rules in initially undifferentiated networks undergoing spontaneous activity (van Heijst et al., 1998; Petersson et al., 2003; Marques et al., 2013). Together with models demonstrating the feasibility of trial-and-error learning (Raphael et al., 2010) this evidence suggests that acquisition of appropriately tuned neural circuits in the spinal cord is possible.

Further work is needed to investigate the exact role of the different feedback modalities (position, velocity, and force) and interneurons in the accommodation of interaction torques in model spinal circuits. Given that the organization of the human spinal cord in reality is significantly more complicated than modeled here, and our substantial yet incomplete knowledge regarding its structure and functionality, an interesting avenue to explore would be to study the complete ensemble of spinal models that fill in unknown data and conform with behavioral and neurophysiological data. Such a methodology has been applied, for example, to study the mechanism underlying klinotaxis in *C. elegans* and to propose experiments that can distinguish between different hypotheses regarding its neural implementation (Izquierdo and Beer, 2013).

In conclusion, the work presented here demonstrates the feasibility of equilibrium point control for multijoint reaching movements subject to varying intersegmental loads. The model shows that internal models and predictive compensation of such loads are not required for the range of movements studied here. But it does not allow us to refute the possibility that such mechanisms are indeed used by the CNS for this or other purposes. The model also indicates that EP style control of reaching movements is dependent on a mapping of desired movement kinematics to control parameters and on the appropriate self-organization of spinal circuitry. We do not propose that it is the sole, or even most central, function of spinal circuitry to implement equilibrium point control, or to coordinate different muscles for interaction torque accommodation. Indeed, while other models not incorporating spinal interneurons might also be able to accommodate interaction torques to some extent, reflex circuitry in the spinal cord in conjunction with central modulation may allow for greater flexibility in the execution of a given class of movements (such as adaptation to varying energy, speed and accuracy trade-offs), and may underlie the ability to perform different classes of mechanical action (such as control of position, force or stiffness) as and when needed.

## Funding

This work is funded by the project “eSMCs: Extending Sensorimotor Contingencies to Cognition,” FP7-ICT-2009-6 no: 270212.

### Conflict of interest statement

The authors declare that the research was conducted in the absence of any commercial or financial relationships that could be construed as a potential conflict of interest.
