# Supplementary material for: Spinal circuits can accommodate interaction torques during multijoint limb movements
Source: Front Comput Neurosci. 2014 Nov 11;8:144. doi: 10.3389/fncom.2014.00144 (PMC4227517; doi:10.3389/fncom.2014.00144)
Supplement: Supplementary file 1 [file Presentation1.PDF]

Frontiers in Computational Neuroscience

Research Article
October 3, 2014

Supplementary Material: Spinal circuits can accommodate interaction torques during multijoint limb movements

Thomas Buhrmann [1], **Ezequiel Alejandro Di Paolo** [1,2,3,*]

[1] *IAS-Research Centre for Life, Mind and Society, UPV/EHU, University of the Basque Country, San Sebastian, Spain*
[2] *Ikerbasque, Basque Foundation for Science, Bilbao, Spain*
[3] *Centre for Computational Neuroscience and Robotics, University of Sussex, Brighton, UK*

Correspondence*:
Ezequiel Alejandro Di Paolo
Department of Logic and Philosophy of Science, Euskal Herriko Unibertsitateko - Universidad del Pais Vasco, Avenida de Tolosa 70, 20080 Donostia/San Sebastián, Spain, ezequiel.dipaolo@ehu.es

Neural and Computational Modeling of Movement Control

1 MODEL PARAMETERS

This is a supplementary document providing a detailed overview of model parameters that were optimised as described in the accompanying article.

Table 1 provides a translation between symbols used for model parameters in the main text (Symbol) and parameter names used in the xml definition below (XML Name).

Symbol	Range	Description	Xml Name
Optimised muscle parameters			
$p_{o,i}$	[0.06, 0.26] m	Distance of insertions from joint	Origin, Insertion
F_{max}	[100, 2000] N	Maximum isometric force	MaxIsoForce
l_0	[0.01, 1.5] l_{max}	Optimum length	Length: Optimal
v_{max}	[8, 12] l_0/s	Maximum contraction velocity	MaxVelocity
$r_{1,2}$	[0.01, 0.05] m	Joint capsule radii	Radius
Optimised neural parameters			
τ	[0.01, 1] s	Time constant	Tau
θ	[-10, 10]	Bias	Bias
w	[-10, 10]	Connection weights	W_{ij} (from neuron i to j)
k	[0.1, 10]	Slope of transfer function	Slope
Optimised threshold control (λ-model) parameters			
k_p	[0, 6]	Position feedback gain	PosGain
k_v	[0, 0.5]	Velocity feedback gain	VelGain
k_d	[0, 1]	Damping gain	DmpGain
λ^{co}	[0, 0.3]	Open-loop cocontraction	
Fixed parameters			
k_{sh}, k_{le}	0.25	Hill-function curvature	Shortening, Lengthening
k_{max}	1.5	Maximum eccentric force	Asymptote
k_m	2	Hill slope multiplier at v=0	Slope
β_{ac}	0.04 s	Muscle activation time scale	Hard-coded
β_{de}	0.07 s	Muscle deactivation time scale	Hard-coded
δ	0.025 s	Proprioceptive feedback delay	FeedbackDelay

The following is a full definition of the arm model listing all optimised and fixed parameter values (rounded) in XML format. Note that for some synaptic strengths absolute values are displayed. The correct sign (exhibitory or excitatory) is hard-coded and provided in the main text. Also, for neural time constants (Tau) inverse values are displayed (1/Tau).

```xml
<Arm>
    <- Virtual EP trajectory duration as fraction of movement duration ->
    <EpDuration>0.446</EpDuration>

    <- Arm Geometry: units in m, kg ->
    <UpperArm Length="0.33" Mass="2.52" Inertia="0.02287"/>
    <LowerArm Length="0.32" Mass="1.3" Inertia="0.0111"/>

    <- Joints: units in degress, m ->
    <Shoulder UpperLimit="160" LowerLimit="0" Locked="0" Radius="0.0432"/>
    <Elbow UpperLimit="160" LowerLimit="0" Locked="0" Radius="0.0469"/>

    <- Muscle definitions: units in m, N, l0/s ->
    <Muscle Joint="Elbow" Name="ElbowFlexor">
        <Attachment Origin="0.2391" Insertion="0.0781"/>
        <MaxIsoForce>138.388</MaxIsoForce>
        <MaxVelocity>11.5859</MaxVelocity>
        <Length Optimal="0.3105" Min="0.1678" Max="0.3364"/>
        <HillParameters Shortening="0.25" Lengthening="0.25" Asymptote="1.4" Slope="2"/>
    </Muscle>
    <Muscle Joint="Elbow" Name="ElbowExtensor">
        <Attachment Origin="0.2391" Insertion="0.0781"/>
        <MaxIsoForce>138.388</MaxIsoForce>
        <MaxVelocity>11.5859</MaxVelocity>
        <Length Optimal="0.4473" Min="0.3364" Max="0.4675"/>
        <HillParameters Shortening="0.25" Lengthening="0.25" Asymptote="1.4" Slope="2"/>
    </Muscle>
    <Muscle Joint="Shoulder" Name="ShoulderFlexor">
        <Attachment Origin="0.2536" Insertion="0.0605"/>
        <MaxIsoForce>1522.92</MaxIsoForce>
        <MaxVelocity>11.1522</MaxVelocity>
        <Length Optimal="0.359" Min="0.198" Max="0.3342"/>
        <HillParameters Shortening="0.25" Lengthening="0.25" Asymptote="1.4" Slope="2"/>
    </Muscle>
    <Muscle Joint="Shoulder" Name="ShoulderExtensor">
        <Attachment Origin="0.2537" Insertion="0.0605"/>
        <MaxIsoForce>1522.92</MaxIsoForce>
        <MaxVelocity>11.1522</MaxVelocity>
        <Length Optimal="0.4771" Min="0.3342" Max="0.4551"/>
        <HillParameters Shortening="0.25" Lengthening="0.25" Asymptote="1.4" Slope="2"/>
    </Muscle>

    <- Spinal circuit innervating elbow muscles ->
    <- "Ag" and "An" refer to neurons innervating flexor and extensor respectively ->
    <Reflex Agonist="ElbowFlexor" Antagonist="ElbowExtensor" FeedbackDelay="0.025">
        <Spindle> <- lambda-model ->
            <PosGain Ag="0.6916" An="0.6916"/>
            <VelGain Ag="0.2695" An="0.2695"/>
            <DmpGain Ag="0.8426" An="0.8426"/>
            <Wspmn Ag="0.7101" An="0.7101"/>    <- Output to motor neuron ->
        </Spindle>
        <IaIn> <- Ia inhibitory interneurons ->
            <Wiaia Ag="0.9805" An="0.9106"/>    <- Reciprocal inhibition ->
            <Wspia Ag="0.7798" An="0.1317"/>    <- Proprioceptive input ->
            <Wrnia Ag="0.4764" An="0.012"/>     <- Renshaw input ->
            <Waia Ag="0.7755" An="0.564"/>      <- Descending desired contraction ->
            <Wiamn Ag="0.456" An="0.3302"/>     <- Output to motor neuron ->
            <Wgoia Ag="-0.6707" An="-0.6707"/>  <- Input from GO signal ->
            <Bias Ag="0.0695" An="0.0272"/>
            <Tau Ag="33.0438" An="87.8991"/>
            <Slope Ag="6.0433" An="6.3943"/>
```

```
        </IaIn>
        <Renshaw> <- Renshaw cells ->
            <Wmnrn Ag="0.6416" An="0.6416"/>    <- Input from motor neuron ->
            <Wrnrn Ag="0.8032" An="0.8032"/>    <- Reciprocal inhibition ->
            <Wrnmn Ag="0.7007" An="0.7007"/>    <- Output to motor neuron ->
            <Wgorn Ag="-0.1034" An="-0.1034"/>  <- Input from GO signal ->
            <Bias Ag="0.0226" An="0.0226"/>
            <Tau Ag="59.7041" An="59.7041"/>
            <Slope Ag="9.0517" An="9.0517"/>
        </Renshaw>
        <IbIn> <- Ib inhibitory interneurons ->
            <Wibib Ag="0.4825" An="0.3393"/>    <- Reciprocal inhibition ->
            <Wglib Ag="0.6603" An="0.2341"/>    <- Force input from Golgi organ ->
            <Wibmn Ag="0.8969" An="0.9805"/>    <- Output to motor neuron ->
            <Wiaib Ag="0.3034" An="0.145"/>     <- Regulation by IaIn ->
            <WisibAg Ag="0.9968" An="-0.4768"/> <- Intersegmental feedback from other flexor ->
            <WisibAn Ag="-0.5583" An="0.4093"/> <- Intersegmental feedback from other extensor ->
            <Wgoib Ag="0.3034" An="0.145"/>     <- Input from GO signal ->
            <Bias Ag="-0.0278" An="0.0234"/>
            <Tau Ag="34.6041" An="35.105"/>
            <Slope Ag="1.9534" An="7.9131"/>
        </IbIn>
        <aMN Wgo="-0.3388799429760041">
            <WeIbinMn Ag="0.7346" An="0.5714"/>     <- Ib reciprocal excitation of antagonist MN ->
            <WisibAgMn Ag="-0.2586" An="0.4342"/>   <- Intersegmental feedback from other flexor ->
            <WisibAnMn Ag="-0.9169" An="-0.6077"/>  <- Intersegmental feedback from other extensor ->
            <Wgomn Ag="-0.3389" An="-0.6077"/>      <- Input from GO signal ->
        </aMN>
    </Reflex>

    <- Spinal circuit innervating shoulder muscles ->
    <Reflex Agonist="ShoulderFlexor" Antagonist="ShoulderExtensor" FeedbackDelay="0.025">
        <Spindle> <- lambda-model ->
            <PosGain Ag="2.5752" An="2.5752"/>
            <VelGain Ag="0.3287" An="0.3287"/>
            <DmpGain Ag="0.7863" An="0.7863"/>
            <Wspmn Ag="0.9968" An="0.9968"/>    <- Output to motor neuron ->
        </Spindle>
        <IaIn> <- Ia inhibitory interneurons ->
            <Wiaia Ag="0.4678" An="0.4926"/>    <- Reciprocal inhibition ->
            <Wspia Ag="0.1812" An="0.262"/>     <- Proprioceptive input ->
            <Wrnia Ag="0.1536" An="0.4642"/>    <- Renshaw inputs ->
            <Waia Ag="0.6328" An="0.0"/>        <- Descending desired contraction ->
            <Wiamn Ag="0.5888" An="0.2956"/>    <- Output to motor neuron ->
            <Wgoia Ag="-0.1726" An="-0.1726"/>  <- Input from GO signal ->
            <Bias Ag="-0.0396" An="0.0045"/>
            <Tau Ag="40.1" An="44.6528"/>
            <Slope Ag="8.8396" An="4.8218"/>
        </IaIn>
        <Renshaw> <- Renshaw cells ->
            <Wmnrn Ag="0.929" An="0.929"/>      <- Input from motor neuron ->
            <Wrnrn Ag="0.3845" An="0.3845"/>    <- Reciprocal inhibition ->
            <Wrnmn Ag="0.9522" An="0.9522"/>    <- Output to motor neuron ->
            <Wgorn Ag="-0.0995" An="-0.0995"/>  <- Input from GO signal ->
            <Bias Ag="-0.0365" An="-0.0365"/>
            <Tau Ag="78.6065" An="78.6065"/>
            <Slope Ag="9.3265" An="9.3265"/>
        </Renshaw>
        <IbIn> <- Ib inhibitory interneurons ->
            <Wibib Ag="0.8116" An="0.5009"/>    <- Reciprocal inhibition ->
            <Wglib Ag="0.0179" An="0.4138"/>    <- Force input from Golgi organ ->
```

```
            <Wibmn Ag="0.8182" An="0.8194"/>    <- Output to motor neuron ->
            <Wiaib Ag="0.3435" An="0.0733"/>    <- Regulation by IaIn ->
            <WisibAg Ag="-0.6229" An="0.3778"/> <- Intersegmental feedback from other flexor ->
            <WisibAn Ag="0.5996" An="0.1512"/>  <- Intersegmental feedback from other extensor ->
            <Wgoib Ag="-0.6487" An="-0.6487"/>  <- Input from GO signal ->
            <Bias Ag="0.096" An="-0.041"/>
            <Tau Ag="36.2238" An="97.3991"/>
            <Slope Ag="0.1003" An="5.0746"/>
        </IbIn>
    </Reflex>

    <- Coactivation components of control signal (lambda^co) ->
    <OpenLoopActivations>
        <- Movement W1 ->
        <Value>0.0753501</Value>    <- Elbow flexor ->
        <Value>0.299998</Value>     <- Elbow extensor ->
        <Value>0.172712</Value>     <- Shoulder flexor ->
        <Value>0.279667</Value>     <- Shoulder extensor ->

        <- Movement R1 ->
        <Value>0.154564</Value>
        <Value>0.00711234</Value>
        <Value>0.21512</Value>
        <Value>5.35819e-05</Value>

        <- Movement W2 ->
        <Value>0.0618431</Value>
        <Value>0.280571</Value>
        <Value>0.140614</Value>
        <Value>0.224172</Value>

        <- Movement R2 ->
        <Value>0.225695</Value>
        <Value>0.123521</Value>
        <Value>0.232145</Value>
        <Value>0.0119114</Value>
    </OpenLoopActivations>
</Arm>
```
